# Aging Negatively Impacts DNA Repair and Bivalent Formation in the *C. elegans* Germ Line

**DOI:** 10.3389/fcell.2021.695333

**Published:** 2021-08-04

**Authors:** Marilina Raices, Richard Bowman, Sarit Smolikove, Judith L. Yanowitz

**Affiliations:** ^1^Department of Obstetrics, Gynecology and Reproductive Sciences, Magee-Womens Research Institute, University of Pittsburgh School of Medicine, Pittsburgh, PA, United States; ^2^Department of Biology, The University of Iowa, Iowa City, IA, United States; ^3^Department of Developmental Biology, Microbiology and Molecular Genetics, Hillman Cancer Center, University of Pittsburgh School of Medicine, Pittsburgh, PA, United States

**Keywords:** DSB repair, *C. elegans*, germline, RAD51, RPA, aging, meiosis

## Abstract

Defects in crossover (CO) formation during meiosis are a leading cause of birth defects, embryonic lethality, and infertility. In a wide range of species, maternal aging increases aneuploidy and decreases oocyte quality. In *C. elegans* which produce oocytes throughout the first half of adulthood, aging both decreases oocytes quality and increases meiotic errors. Phenotypes of mutations in genes encoding double-strand break (DSB)-associated proteins get more severe with maternal age suggesting that early meiosis reflects a particularly sensitive node during reproductive aging in the worm. We observed that aging has a direct effect on the integrity of *C. elegans* meiotic CO formation, as observed by an increase of univalent chromosomes and fusions at diakinesis, with a considerable increase starting at 4 days. We also characterize the possible causes for the age-related changes in CO formation by analyzing both steady-state levels and kinetics of the ssDNA binding proteins RPA-1 and RAD-51. Profound reductions in numbers of both RPA-1 and RAD-51 foci suggests that both DSB formation and early meiotic repair are compromised in aging worms. Using laser microirradiation and γ-irradiation to induce exogenous damage, we show specifically that recruitment of these homologous recombination proteins is altered. Repair defects can be seen in two-and-one-half day-old adults making the loss of germline repair capacity among the earliest aging phenotypes in the worm.

## Introduction

It has been shown that maternal age negatively impacts fertility across species. Particularly in humans, female reproductive capacity declines dramatically after the mid-30 s making it the earliest age-related decline that humans experience (Hassold and Hunt, 2001). A major cause of this reproductive decline is the increasing incidence of oocytes with chromosomal abnormalities, which leads to miscarriage and congenital defects. As more women opt to have children later in life, addressing the issue of female reproductive aging has become increasingly important. Recently, the nematode *Caenorhabditis elegans* has been developed as a model to study reproductive capacity decline with age (Hughes et al., 2007; Andux and Ellis, 2008; Luo et al., 2010; Quesada-Candela et al., 2021).

During meiosis, crossovers (COs), the physical exchange of genetic material between homologs, together with the action of cohesin proteins, ensure proper alignment and segregation of the chromosomes at meiosis I. Across species, age-related loss of cohesin is associated with increased chromosomal non-disjunction (Hodges et al., 2005; Subramanian and Bickel, 2008). In addition, specific CO configurations, specifically those with too few, too many, or misplaced COs (near centromeres and telomeres), are acutely sensitive to maternal age, although they can be segregated appropriately in younger animals (Hodges et al., 2005; Lamb et al., 2005). Historically, numerous studies documented changes in recombination rates in response to external factors, including maternal age (for example, Stern, 1926; Rose and Baillie, 1979; Hussin et al., 2011). As more chromosome-wide techniques have evolved, changes in crossover positioning have also been reported (Lim et al., 2008; Saini et al., 2020). Nevertheless, our understanding of the factors that drive these age-related changes in CO distribution and frequency is still limited.

In species which produce oocytes throughout adult life, defects in DSB induction and CO repair may further impair oocyte quality in older animals. COs are induced by the formation of meiotic double-strand breaks (DSBs) which are repaired by homologous recombination (HR) with specializations for meiosis. DSBs are induced by the evolutionarily conserved topoisomerase VI-like Spo11 (Keeney et al., 1997) in concert with accessory factors that influence the timing, placement, and extent of break formation. Endonucleolytic cleavage by the MRX/N complex releases Spo11 bound to short oligonucleotides and generates 3′ single-stranded DNA (ssDNA) tails (Neale et al., 2005) that are rapidly resected and bound by the ssDNA binding protein complex RPA, protecting them from degradation. RPA is replaced by RAD51 and/or DMC1 which form (Gasior et al., 1998) filaments then can invade the homologous double stranded-DNA of either the sister chromatid or homologous chromosome. This single end-invasion can go on to form double Holliday Junctions (dHJ) (Shinohara et al., 1992; Sung, 1994), a subset of which are resolved to form COs (Schwacha and Kleckner, 1995). COs, together with cohesion, connect the homologous chromosomes, enabling their biorientation toward opposite spindle poles in metaphase I (reviewed in Sansam and Pezza, 2015).

The events of meiosis are tightly regulated to ensure both that each chromosome attains a crossover and that excessive DSBs are repaired and not passed along to offspring. Accordingly, non-HR pathways can step to repair DSBs if HR is ineffective. These include the classical non-homologous end joining (cNHEJ) pathway, microhomology-mediated end jointing (MMEJ), and single-strand annealing (SSA) (Macaisne et al., 2018). In *C. elegans*, DSB-promoting factors HIM-5 and CEP-1/p53 actively repress NHEJ functions allowing preferential use of HR (Mateo et al., 2016), intimating that alternative pathways can be available for DSB repair in early meiosis and are not simply late-acting salvage pathways that eliminate residual breaks.

Aging has a direct effect on the integrity of *C. elegans* meiotic CO formation as an increase in achiasmate chromosomes at diakinesis was observed at day 5 of adulthood, half-way through the worm’s reproductive span (Luo et al., 2010; Meneely et al., 2012; Stamper et al., 2013; Achache et al., 2020). Also, it has been reported that maternal age exacerbates CO defects associated with mutations in SPO-11 accessory factors including *dsb-2* and *him-5* (Meneely et al., 2012; Stamper et al., 2013). This suggests that either the DSB break formation or repair machineries (or both) might be inherently sensitive to maternal age. However, the underlying cause(s) of this aging phenotype is not understood. In this study we tested the hypotheses that DSB formation and repair are impaired in aging. We analyzed the effect of maternal age on the recruitment of different HR proteins. To address this, we utilized two irradiation techniques (laser microirradiation and γ-irradiation) to induce exogenous damage in meiotic nuclei of young and aged worms (Harrell et al., 2018; Koury et al., 2018; Mahadevan et al., 2019). We observed that the total numbers of RPA-1 and RAD-51 foci were reduced by maternal age which results from both a decrease in HR-proficient DSBs and attenuated capability to recruit RPA-1 and RAD-51.

## Materials and Methods

### Strains and Genetics

All strains were derived from the wild-type Bristol strain N2 and were cultivated at 20°C under standard conditions. Abbreviated names and full genotypes of the strains used in this study are listed in Table 1.

**TABLE 1:** *C. elegans* stocks used in this study.

Stock name	Genotype	Abbreviated name in manuscript
N2	*C. elegans* var Bristol (N2).	Wild type
VC531	*rad-54(ok615)* I*/hT2 [bli-4(e937) let-?(q782) qIs48]* (I;III)	*rad-54*
SSM476	*rpa-1(iow92[OLLAS::rpa-1])* II	*OLLAS::rpa-1*
SSM473	*iowSi8[pie-1p::gfp1-10::him-3 3UTR + Cbr-unc119(+)]*, *rpa-1(iow89[gfp11::rpa-1])* II; unc-119(ed3)*	*gfp::rpa-1*
QP1118	*meIs8[unc-119(+) pie-1^promoter^::gfp::cosa-1]* II	*gfp-1::cosa-1*
SSM639	*iowSi8[pie-1p::gfp1-10::him-3 3UTR + Cbr-unc119(+)]*, *rpa-1(iow89[gfp11::rpa-1])* II*; unc-119(ed3)* III*;; spo-11(ok79)* IV*/nT1 [unc-?(n754) let-?]* (IV;V)	*gfp::rpa-1; spo-11*
QP1266	*meIs8[unc-119(+) pie-1^promoter^::gfp::cosa-1]* II*; spo-11(me44)* IV*/nT1 [unc-?(n754) let-? qIs50]* (IV;V)*	*gfp::cosa-1; spo-11*
TG2228	*polq-1(tm2026)* III	*polq-1*
FX1524	*cku-70(tm1524)* III	*cku-70*
AV106	*spo-11(ok79)* IV*/nT1 [unc-?(n754) let-?]* (IV;V)	*spo-11*

*The unc-119 gene is used as a selection marker for the transgenes. Prior studies of the cosa-1 strain suggest it does not appear to interfere with crossover formation (Yokoo et al., 2012).

### Chromosome Morphology Analysis of Diakinesis Oocytes

The numbers of DNA bodies present in diakinesis oocytes were assessed in intact adult hermaphrodites at days 1 through 10 of adulthood. Animals were picked as L4 larval and transferred each day until the cessation of egg-laying. Adults were fixed in Carnoy’s fixative solution (60% ethanol, 30% chloroform, and 10% glacial acetic acid) and stained with 4′,6-diamidino-2-phenylindole (DAPI) (10 mg/ml stock diluted 1:50,000 in 1X Phosphate-buffered saline (PBS) for 15 min. in a humidity chamber. Stain was removed and worms were mounted in Prolong Gold with DAPI, cured overnight at room temperature, and stored at 4°C prior to imaging on a Nikon A1r confocal microscope. Diakinesis images were procured as 0.2μm per plain Z-stacks and visualized using Volocity 3-D Imaging Software (PerkinElmer Corp, now owned by QuorumTechnologies).

### Immunostaining and Analysis of RAD-51 Foci

Gonads were dissected from 1-1 and 4-days adults in 1X sperm salts (50 mM PIPES pH 7.0, 25 mM KCl, 1 mM MgSO_4_, 45 mM NaCl, and 2 mM CaCl_2_) with 1 mM levamisole and fixed in 1% paraformaldehyde diluted in 1X sperm salts for 5 min in a humid chamber. Slides were then frozen on a metal block on dry ice for at least 10 min prior to flicking off the cover slip and immersing in methanol at −20° C for 2 min followed by 5 s in acetone at 4°C. Slides were then washed in PBSTB [1XPBS with 0.1% Tween and 0.1% bovine serum albumin (BSA)], and incubated overnight at 4°C with primary antibody: rabbit anti-RAD-51 generated against peptide ASRQKKSDQEQRAA by Genscript for the Smolikove lab (Alleva et al., 2019), 1:30,000 in PBSTB). The next day, slides were washed 3X in PBSTB for 10 min and incubated with secondary antibody (goat anti-rabbit Alexa 568, 1:2,000 in PBSTB) for 2 h at room temperature in the dark. Slides were then washed 2 X 10 min in PBSTB, and 1 X 10 min with DAPI (10 mg/ml stock diluted 1:50,000 in 1X PBS). Slides were mounted in Prolong Gold with DAPI and put in the dark to cure overnight before imaging.

Three-dimensional (3D) images of the whole germ lines were taken using a Nikon A1r confocal microscope with 0.2 μm step size and analyzed using Volocity 3D software (PerkinElmer). For wild-type worms, we divided the leptotene, zygotene, and pachytene regions into six zones and counted RAD-51 foci in every nucleus for a minimum of three germ lines/age. For *rad-54* mutants, we counted RAD-51 foci in late pachytene nuclei for at least three germ lines/age. For irradiated worms, 15 mid-pachytene nuclei were analyzed.

### Analysis of RPA-1 Foci

Gonads of *gfp::rpa-1; spo-11* worms were dissected from 1– to 4-days adults in M9 with 1 mM levamisole. Slides were then freeze-cracked and immersed in cold 100% EtOH for 2 min followed by 20 min in 4% paraformaldehyde/M9 in a humidity chamber. Slides were then washed in PBSTB 1×10 min, incubated with DAPI 1×10 min and washed again. After a quick dry, slides were mounted in Prolong Gold with DAPI.

### Analysis of GFP::COSA-1 Foci

*gfp::cosa-1* and irradiated *gfp::cosa-1; spo-11* worms were dissected in 1X sperm salts as described above. Slides were immediately freeze-cracked and immersed in 100% cold ethanol for 1 min, and fixed in 4% paraformaldehyde/1X sperm salts again for 10 min. Slides were washed 2X10 min in PBSTB, stained with DAPI in 1XPBS for 10 min followed by one wash with PBSTB for 10 min. Slides were mounted in Prolong Gold with DAPI. Images were acquired and analyzed as described above. GFP::COSA-1 foci in late pachytene nuclei (4 rows of nuclei before diplotene) were counted in at least five germ lines/age.

### Western Blotting

For each age, 300 worms were transferred into a glass conical tube and washed thrice with 1X M9 buffer (22 mM KH_2_PO_4_, 42 mM Na_2_HPO_4_, 85.5 mM NaCl, 1 mM MgSO_4_). The remaining liquid was removed, and the worm pellet was transferred to a 1.5 ml tube and flash frozen in liquid nitrogen. Pellets were thawed on ice, mixed with an equal volume of Laemmli Sample Buffer (Bio-Rad #161-0737) with 5% b-mercaptoethanol (Amresco M131), sonicated in a water bath for 2 min, heated at 95°C for 10 min, then spun in a tabletop centrifuge for 5 min at maximum speed. Samples were resolved by 12% PAGE (TGX FastCast, Bio-Rad) and transferred to nitrocellulose in 20% ethanol. The membrane was blocked in 5% non-fat milk/TBST (50 mM Tris-HCl pH 7.4, 150 mM NaCl, and 0.1%Tween-20) for 1 h at RT and incubated in OLLAS-tag antibody, pAb, rabbit (GenScript, A01658, 1μg/ml in 5% milk/TBST) or mouse α-E7 (tubulin, Developmental Studies Hybridoma Bank, 1:2,000 in 5% milk/TBST) ON at 4°C. The next day the membranes were washed in TBST and incubated in α-rabbit-HRP or α-mouse-HRP (1:2,000 in 5% milk/TBST) for 2 h at room temperature. Products were visualized by enhanced chemiluminescence (ECL) according to the manufacturer’s instructions.

### γ-Irradiation

1– and 4– days adult worms were exposed to 10Gy of γ-irradiation using a ^137^Cs source (Gammacell1000 Elite; Nordion International). RAD-51 immunostaining was performed as described above on gonads dissected and fixed at 25 min, 4 and 8 h post-IR. RPA-1 detection was assessed at 25 min and 4 h post-IR and GFP::COSA-1 analysis were performed at 8 h post-IR.

### Laser Microirradiation, Live Imaging, and Fixation

We followed the protocol outlined in Harrell et al. (2018) with the following modifications: for the live imaging experiments, five worms were imaged at a time at 2 min intervals for 45 min. Z stacks of 10 images at 1μm stages were taken at each interval. For the fixed sample experiments five worms were placed on a live imaging slide, microirradiated, recovered to an NGM plate until each specified time point, then placed in M9 on frosted microscope slides. The M9 was removed and the worms were fixed in 100% EtOH and mounted with VectaShield.

### Endogenous RPA-1 Intensity Measurements

*gfp::rpa-1* adult worms were grown to 1, 2, 3, and 4 days old. Their gonads were dissected on charged slides, frozen at −80°C, and placed in −20°C EtOH for 1 min then mounted with 10 μl M9-DAPI and VectaShield. Whole intact gonads were imaged on the DeltaVision wide-field fluorescence microscope (GE Lifesciences) in seven 512 × 512 pixel zones with 100x/1.4 NA oil Olympus objective from the proximal pre-meiotic tip to the distal late pachytene phase of prophase I. Each image was the center slice of the nuclei in the upper section of the syncytial tube in both the DAPI (blue) and FITC (GFP) fluorescent channels. Measurement of the intensity of each nucleus was taken in FIJI in each zone in the FITC channel and corrected to cytoplasmic background.

### Counting Recruitment Regions

Recruitment regions were counted in FIJI by visual inspection of each Z-slice of each time interval. A recruitment region was determined to be in response to microirradiation if it appeared in a location that did not contain a localization of RPA-1 fluorescence in the initial control image. Clusters were differentiated from foci when more than one distinct point of fluorescence was present but not separate from each other.

### Statistical Analyses

*All statistics were performed* using GraphPad Prism 9. For all pairwise comparisons presented, the Mann-Whitney *U*-test was performed. For all binary data comparisons, the Fisher’s Exact test of independence was used.

### Ethics Statement

No studies in this paper required approval by institutional review boards.

## Results

### Aging Increases Meiotic Defects in *C. elegans*

**FIGURE 1:**
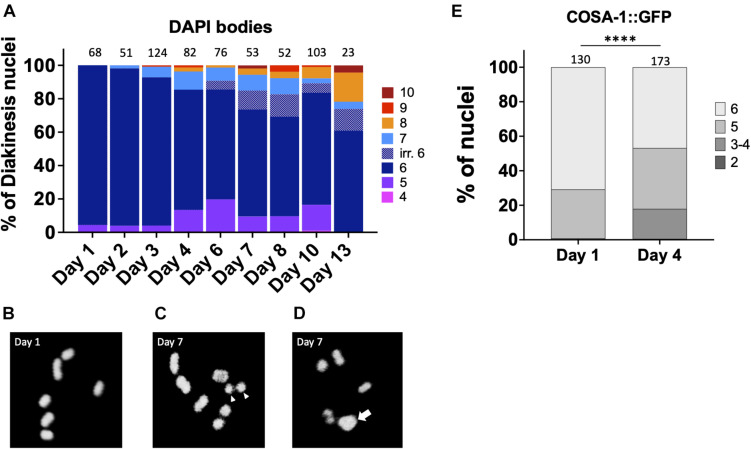
Chiasmata formation is compromised in aging germ lines. Crossover formation is assessed by the number of DAPI-staining bodies at diakinesis. **(A)** Quantification of DAPI-bodies in N2 diakinesis nuclei from the indicated ages. **(B–D)** Representative DAPI-stained images of young **(B)** and aged **(C,D)** N2 worms showing bivalents (day 1), “separated bivalents” (arrowheads) and fusion (arrow). **(E)** Percent of late pachytene-stage nuclei containing the indicated number of COSA-1 foci in *gfp::cosa-1* worms. Numbers on top of bars indicate the total number of nuclei analyzed per age. Statistical significance was determined using Chi-square test. ^****^p < 0.0001.

Multiple lines of evidence indicate that fertility in *C. elegans* declines with maternal age (Hughes et al., 2007; Luo et al., 2010). In both unmated and mated hermaphrodites, days 4–5 of adulthood marks a decline in reproductive capacity that is accelerated in the unmated animals due to lack of sperm (Luo et al., 2010). This timepoint is marked by increases in both inviable eggs and male offspring (Hughes et al., 2007; Luo et al., 2010) which can both be explained by the concomitant increase in CO errors seen in diakinesis oocytes. In order to determine when CO defects are first observed, we analyzed the integrity of chromosomes in N2 diakinesis nuclei throughout adulthood. In young adult animals (day 1), six bivalents were detected at diakinesis by fluorescence microscopy, corresponding to the six pairs of homologous chromosomes each held together by a chiasma (Figures 1A,B). A small fraction of nuclei (4%) showed 4 bivalents and 1 larger mass of DNA (Figure 1D), a configuration that arises in repair mutants and corresponds to a chromosomal fusion (Martin et al., 2005) but may result from two closely apposed chromosomes (for simplicity, we refer to these as fusions). Since *C. elegans* chromosomes are holocentric, fusion chromosomes would be stably transmitted during meiosis and the subsequent mitotic embryonic divisions, explaining the near 100% hatching rates of eggs. The small fraction of fusions was maintained in 2- and 3-days-old adults. However, after this timepoint, nuclei with five bivalents and two univalents were also apparent, indicating that one pair of chromosomes failed to achieve a CO. As the worms aged, meiotic defects worsened. In 4-day-old adults, ∼15% of the analyzed diakinesis showed nuclei with achiasmate chromosomes and ∼14% exhibited fusions. Since day 4 of adulthood corresponds to the time when meiotic errors increase substantially (Figure 1A) and when egg production begins to decrease (Hughes et al., 2007; Luo et al., 2010), we chose this timepoint as the “aged worms” for the remainder of the experiments described herein.

After day 4, meiotic errors continue to increase until reproductive senescence sets in at day 10. Curiously, older animals (>6-days-old) showed some irregular bivalents where the homologous chromosomes appeared to a be separating from one another (Figure 1C). Similar separating bivalents were recently reported for aged worms grown at 25°C, and the distinct univalent masses each contained the crossover marker, COSA-1 (Achache et al., 2020). At timepoints after egg-laying has ceased, days 10 and day 13, there remain a surprisingly large fraction of diakinesis oocytes with 6 DAPI bodies meaning that chromosomal abnormalities are not driving reproductive senescence.

To corroborate the impact of age on CO formation, we also examined GFP::COSA-1 foci, a cytological marker of CO commitment (Yokoo et al., 2012). As shown in Figure 1D, in 1 day-old adults, 5–6 COSA-1 foci are seen in the last 4 rows of nuclei prior to diplotene, consistent with prior studies (Yokoo et al., 2012; Machovina et al., 2016; Woglar and Villeneuve, 2018). By contrast, in day 4 adults, nearly 20% of nuclei had only 3 or 4 COSA-1 foci indicative of an impairment in CO formation. This decrease in COSA-1 foci could result from the combined effects of fewer DSBs, altered repair kinetics, as well as impaired recruitment of CO designation and commitment factors. Overall, these data support the conclusion that fewer COs are observed in older animals.

### The Levels of Homologous Recombination Competent Double-Strand Breaks Decrease With Maternal Age

**FIGURE 2:**
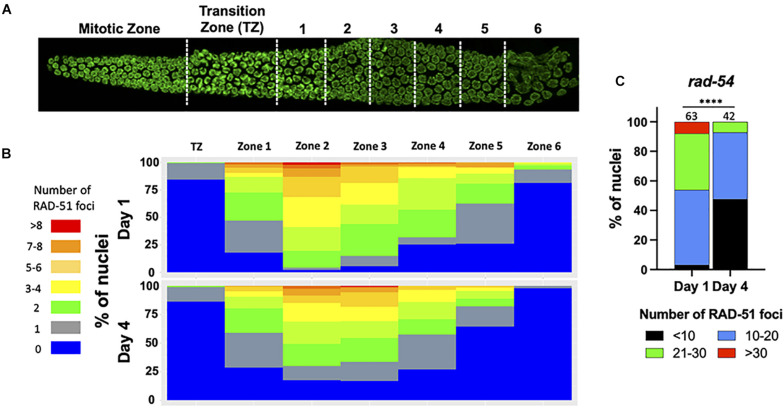
Fewer RAD-51 foci in meiotic nuclei from aged mothers. **(A)** DAPI-stained *C. elegans* germ line showing the regions in which RAD-51 foci were quantified. Each zone corresponds to a particular meiotic stage: Transition zone (TZ) corresponds to leptotene and zygotene, Zones 1 and 2 to early pachytene, Zones 4 and 5 to middle pachytene and Zones 5 and 6 to late pachytene. **(B)** The percentage of nuclei in each zone containing the indicated number of RAD-51 foci. Four gonads per age in N2 worms were quantified. **(C)** Quantification of RAD-51 foci in the *rad-54(ok615)* mutant background. Shown is the percentage of nuclei in late pachytene nuclei containing the indicated number of RAD-51 foci. Numbers on top of bars indicate the total number of nuclei analyzed per age. Statistical significance was determined using Chi-square test. ^****^p < 0.0001.

The increase in univalent (non-CO chromosomes) with maternal age could be explained by the decrease of meiotic-specific DSB formation. Therefore, to determine if DSB formation is itself age-sensitive, we took advantage of RAD-51 immunostaining which marks early intermediates of homologous recombination repair, forming foci. Prior studies had shown that greater than 95% of meiotic DSBs can be marked with RAD-51 (Mets and Meyer, 2009). In day 1 adults, we observed the RAD-51 localization pattern expected for wild type *C. elegans* meiosis: RAD-51 foci began to appear during leptotene/zygotene, became numerous by mid- pachytene, and disappeared by late pachytene (Figures 2A,B), indicative of efficient DSB repair (Alpi et al., 2003; Mateo et al., 2016). On day 4 of adulthood, the same relative pattern was observed, although the total number of foci was reduced in each region of the early prophase germ line (Figure 2B). This was seen most strikingly by the increase in the number of nuclei in the mid-pachytene regions that have zero RAD-51 and the decrease in nuclei with > 6 RAD-51 foci. A decrease in steady-state RAD-51 levels was also reported (Achache et al., 2020). In *spo-11* mutant animals where meiotic DSBs are not made, few to no breaks are seen in Day 1 animals whereas a single RAD-51 focus can be seen in up to half of pachytene nuclei (Supplementary Figure 1), suggesting that if anything, the RAD-51 observed in wild-type 4 day-old adults is an over-representation of meiotically-induced breaks. These results suggest either that DSB numbers decrease with age, or that DNA repair kinetics change with age, or both.

Because DSBs are not made simultaneously and because RAD-51 is removed from the break sites as DSBs mature into COs, a static snapshot of the germ line cannot report the total number of DSBs that are made and undergo CO recombination. To assess total DSB numbers, we took advantage of the *rad-54* mutant in which HR is stalled at a step after the recruitment of RAD-51 to resected DSBs, effectively trapping RAD-51 at all of the sites undergoing HR repair (Meneely et al., 2012). Consistent with prior studies, we observed that nearly 50% of day 1 nuclei accumulated between 20 and 40 RAD-51 foci, more than enough DSBs to ensure a CO on each chromosome (Figure 2C). In contrast, on day 4 of adulthood, > 95% of the nuclei analyzed showed fewer than 20 RAD-51 foci, indicating that the total number of HR-competent breaks decreased with maternal age (Figure 2C). We previously showed that the *him-5(ok1896)* null mutant which fails to achieve COs on the X chromosome contains an average of 9.9 RAD-51 foci/nucleus (Meneely et al., 2012). By inference, the nuclei in older animals with fewer than 10 foci are likely to experience deficits in CO formation.

### Homologous Recombination Is Less Efficient in Older Adult Germ Cells

Since the studies above suggest that DSB levels are decreased in older animals and/or may be repaired by non-HR mechanisms, we wanted to directly analyze HR efficiency in meiotic germ cells. To do this, we took advantage of a previously reported assay to examine repair kinetics after γ-radiation (IR)-induced DSBs (Li and Yanowitz, 2019). Post-IR, we assayed the dynamics of RPA-1 and RAD-51. RPA-1 is the ssDNA-binding protein that is the first to coat the resected DNA (Caldwell and Spies, 2020). Importantly, RPA-1 levels do not decrease with maternal age (Supplementary Figure 2B). Like RAD-51, RPA-1 is observed as chromatin-associated foci in fixed samples. These studies were performed in the *spo-11* mutant background to eliminate the signal from endogenous meiotic breaks. Previous work determined that 10Gy IR is able to produce ∼20 DSBs per meiotic nucleus, which is sufficient to ensure a CO on each chromosome and is near the endogenous number of meiotic DSBs per nucleus (Mets and Meyer, 2009).

**FIGURE 3:**
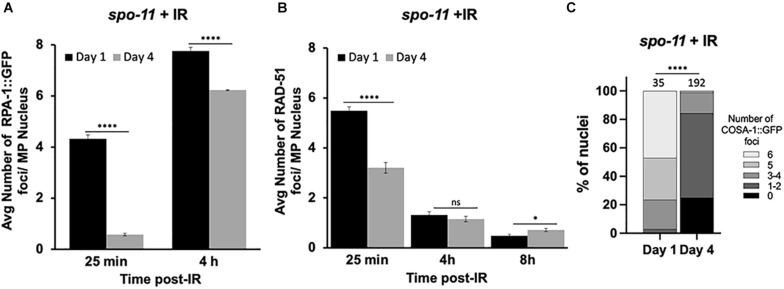
Dynamics of RPA-1 and RAD-51 are altered in aging, meiotic germ cells. *spo-*11 mutant worms at day 1 and day 4 of adulthood were exposed to 10Gy of γ-irradiation. **(A)** Average number of GFP::RPA-1 or **B**) or RAD-51 in mid-pachytene nuclei at indicated timepoints post-exposure. A total of 10 worms per age/timepoint were analyzed in *gfp::rpa-1; spo-11* **(A)** or *spo-11* **(B)** worms. **(C)** Percent of late pachytene nuclei containing the indicated number of COSA-1 foci in *gfp::cosa-1, spo-11* worms at 8 h post-exposure. Statistical significance was determined using Chi-square test. ns *p* > 0.1, ^∗^*p* < 0.05, ^****^p < 0.0001.

At the earliest time point post-IR (25 min), 99% of the day 1-adult, pachytene nuclei recruited RPA-1 to at least one site, with an average of ∼4.3 foci/nucleus. In contrast, 64% of day 4-adult nuclei failed to recruit RPA-1. Although at 4 h post-irradiation both groups of worms showed elevated numbers of RPA-1 foci, the recruitment of RPA-1 was significantly lower in older animals (Figure 3A and Supplementary Figure 3). To verify that the expression of GFP::RPA-1 is not reduced over time, thus leading to the reduction on RPA-1 recruitment, we performed measurements of the intensity of the nuclear GFP signal in young adults and 2, 3, and 4 day adults. These experiments were performed on fixed samples to allow analysis at higher resolution. We observed no decrease in overall GFP signal throughout aging (Supplementary Figure 2). Together these results suggest that aged animals are defective in recruitment of RPA-1 to DSB sites.

Similar defects in initial recruitment to DNA lesions are observed for RAD-51. At 25 min post-IR, significantly fewer RAD-51 foci are seen in day 4 compared to day 1 adults. RAD-51 foci are diminished in both groups at 4 and 8 h post-IR (Figure 3B). However, RAD-51 loss appears slower in 4-day-old adults, as more foci were observed at 8 h post-IR in the day 4 compared to the day 1 adults. While we do not understand the basis for the differences in the patterns of RPA-1 and RAD-51, both sets of data point to altered kinetics of HR repair in older animals.

We also quantified the ability of IR-induced breaks to be converted to CO-committed intermediates by quantifying the recruitment of the COSA-1 to chromosomal foci in late pachytene nuclei (Yokoo et al., 2012). As shown in Figure 3C, we observed a remarkable reduction in GFP::COSA-1 foci in the aged animals 8 h post-IR. At this timepoint, nearly half of day 1 nuclei achieved 6 CO- competent foci, whereas almost no day 4 nuclei experienced full COSA-1 levels (Figure 3C). Together with our observations that fewer early HR-intermediates are observed in the older animals, these results suggest that the ability to undergo CO recombination is compromised in older animals.

In addition to HR-mediated repair pathways, DSBs can also be repaired by other mechanisms including classical non-homologous end joining (cNHEJ), micro-homology mediated end joining (MMEJ), single-strand annealing (SSA). Break-induced replication (BIR) has not been shown to work in meiosis. We previously showed that in the absence of RAD-51 proteins, SSA, NHEJ, and MMEJ all contribute to repair in the meiotic germ line and that HIM-5 biases use of SSA over NHEJ and MMEJ, suggesting that active mechanisms inhibit use of these alternative DSB repair pathways (Macaisne et al., 2018). We therefore hypothesized that some of the abnormal chromosomal patterns that we observe in diakinesis oocytes from older animals might be explained if these non-HR pathways were to be used more frequently. To test this, we examined the diakinesis figures in *cku-70* and *polq-1* mutant animals that are impaired in NHEJ and MMEJ, respectively. As shown in Supplementary Figure 4, impairing NHEJ or MMEJ functions has little to no effect in young animals. In contrast, in older animals loss of *cku-70* or *polq-1* increases the number of nuclei with 6 DAPI bodies. Thus, at least a subset of the chromosome morphology defects observed in diakinesis of older animals is explained by the aberrant activation of NHEJ or MMEJ.

### Repair of Complex DSBs Is Severely Impaired in the Aging *C.elegans* Germ Line

Next, we tested whether the age-dependent defects in recruitment of RPA-1 could be aggravated when the DNA damage formed is more complex. Clustered DSBs are forms of complex DNA damage that are more challenging to repair (Nickoloff et al., 2020). Laser microirradiation induces targeted and complex DNA damage that recruits HR proteins and forms characteristic, clustered DSBs (Koury et al., 2018). To examine RPA-1 localization to DSBs in live worms, we utilized the GFP11 tagged RPA-1 strain (Hefel and Smolikove, 2019) described above. In this strain endogenous RPA-1 foci are dim and can be clearly separated during analysis from microirradiation induced foci (Hefel et al., 2021).

**FIGURE 4:**
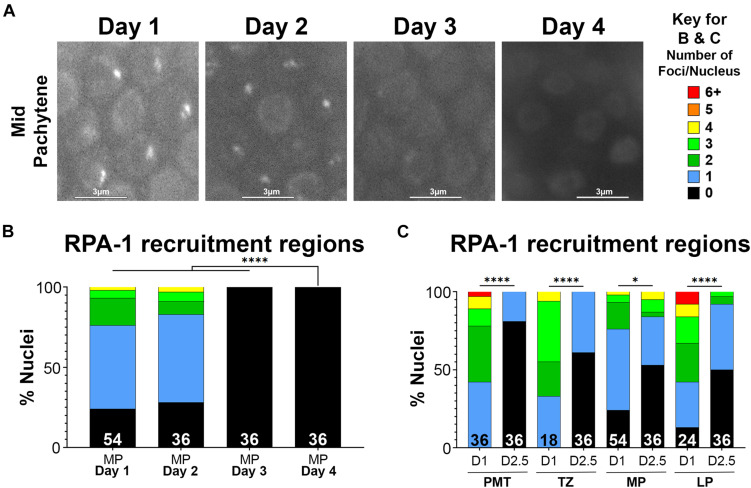
Recruitment of RPA-1 to complex DNA damage decreases with maternal age upon DSB formation. Live imaging of mid-pachytene nuclei post-exposure to microirradiation. **(A)** Left: Representative images from each age of worms examined showing GFP::RPA-1 fluorescence in 20 min post- microirradiation. Images are extracted from the 45-min live-imaging sequence. Scale bars are 3μm. Right: Key for number of recruitment regions represented by each color in panels **(B,C)**. **(B)** Percent of mid-pachytene (MP) nuclei for each age of worm (days post-L4) with the indicated number of GFP::RPA-1 recruitment regions. **(C)** Percent of nuclei in 1-day- and 2.5-day-old adults with the indicated number of GFP::RPA-1 recruitment regions (PMT, pre-meiotic tip; TZ, transition zone; MP, mid-pachytene; LP, late pachytene). Statistical significance was determined using the Fisher’s Exact test and *p*-values are indicated as follows: ns ≥ 0.1234, 0.0332–0.1233 = ^∗^, ≤ 0.0001 = ^****^. Nuclei *n*-values as indicated for each condition.

To test if RPA-1 recruitment to complex DSBs is impaired with aging we exposed worms to microirradiation at days 1, 2, 2.5, 3, and 4 of adulthood and imaged mid-pachytene (MP) nuclei for 45 min (at 2 min intervals) post-exposure. This time frame was determined in our previous studies where we showed that most RPA-1 foci are recruited within the first 30 min in this strain (Hefel et al., 2021). RPA-1 is recruited to microirradiation induced breaks in a pattern of clusters and foci that cannot be resolved by live imaging (Koury et al., 2018) and is collectively referred to here as recruitment regions. Although the number of recruitment regions was no different between 1– and 2-day-old adults, recruitment in the 45 min window was impaired in 2.5-day-old adults and completely abrogated in 3– and 4-day-old adults (Figure 4). To examine if the defects in RPA-1 recruitment were unique to the MP region of the germ line, we performed microirradiation experiments in the other germline regions. We observed similar reductions in RPA-1 recruitment throughout the germ line of aged animals (Figure 4C). These data altogether indicated that despite accumulating in the nucleus, RPA-1 fails to assemble on clustered DNA damage in the same kinetics in aged and non-aged worms.

**FIGURE 5:**
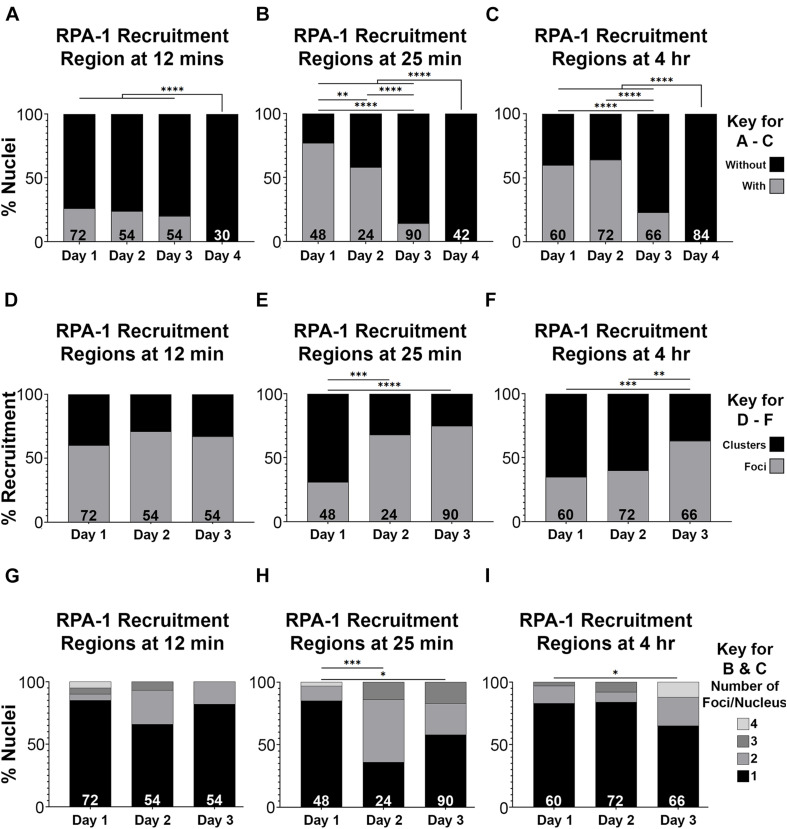
RPA-1 dynamics complex DNA damage attenuates with age. **(A–C)** Percent of mid-pachytene nuclei with RPA-1 recruitment regions out of total number of nuclei 12 min **(A)**, 25 min **(B)** and 4 h **(C)** after microirradiation, in worms at 1, 2, 3, and 4 days post-L4. **(D–F)** Percent RPA-1 recruitment regions appearing as individual foci or as clustered foci per nucleus at 12 min **(D)**, 25 min **(E)** and 4 h **(F)** after microirradiation, in worms at 1, 2, and 3 days post-L4. **(G–I)** Percent of nuclei with the indicated number of RPA-1 recruitment regions per nucleus 12 min **(A)**, 25 min **(B)** and 4 h **(C)** after microirradiation, in worms at 1, 2, and 3 days post L4. Statistical significance was determined using the Fisher’s Exact test and *p*-values are indicated as follows: ns ≥ 0.1234, 0.0332–0.1233 = ^∗^0.0021–0.0331 = ^∗∗^0.0002–0.0020 = ^∗∗∗^, ≤ 0.0001 = ^****^. *N* values as indicated for each condition.

Defects in recruitment of proteins following microirradiation can reflect an abrogation of RPA-1 recruitment or a delay in RPA-1 recruitment to clustered DSBs. Since live imaging data can only be acquired in a limited time window (due to expiration of the worms), analysis in large time scales can only be performed on fixed samples. For this analysis, we microirradiated worms, and then performed gonad dissections at 12 and 25 min, and 4 h timepoints post-microirradiation. We first quantified the percent of nuclei with RPA-1 recruitment regions out of the total number of microirradiated nuclei. 1– and 2-day old adults exhibited maximal recruitment of RPA-1 at the 25 min time point which declined slightly at 4 h (Figures 5A–C). In agreement with our live-imaging data, RPA-1 recruitment to microirradiated nuclei was severally impaired in 3-day-old adults and completely abrogated in 4-day-old adults. These data indicate that aged worms fail to recruit RPA-1 to complex DSBs. Aged worms also exhibited an increase in the proportion of foci over clusters (Figures 5D–F) which is consistent with inhibition of RPA-1 accumulation on microirradiation-induced DNA damage. Aged worms showed a small increase in number of foci per nucleus in later timepoints (Figure 5I, compared to 5G,H). Since for this analysis only nuclei with RPA recruitment regions were scored, this indicates that in the few nuclei in which RPA-1 succeeded in loading to DSBs RPA-1 did not unload in a timely manner.

Altogether our data indicated that, as observed with γ-irradiated worms, microirradiated worms also exhibit defects in RPA-1 recruitment. These defects were more severe in the microirradiated worms, likely due to the complexity of microirradiation induced damage that is more challenging to repair.

## Discussion

While it has been appreciated for decades that oocyte quality diminishes with maternal age, our understanding of the underlying deficits that arise in aged germ cells is still incomplete. We show here that the meiotic processes that control DNA repair and crossover formation deteriorate progressively with maternal age in *C. elegans*. As early as two and one-half days into adult life, just less than one-third of the way through its maximum reproductive period, wild-type worms have profound changes in their ability to recruit DSB-repair proteins to DNA lesions. These defects are the among the earliest harbingers of adult aging in the worm, preceding morphological and physiological somatic changes by several days (Son et al., 2019). In this regard, the oogenic germ line of the adult hermaphrodite may be similar to a human ovary, which also undergoes aging before the rest of the tissues in the body (Hassold and Hunt, 2001).

Since oocytes are made continuously in the worm, we were able to examine how meiotic processes changes in response to age. Using RAD-51 as a surrogate for DSBs, we observed a substantial decrease in HR-competent DSBs in aged germ lines. Since RAD-51 is downstream of resection, a decrease in focus formation could be explained by a *bona fide* decrease in DSB numbers and/or may reflect that DSBs are steered into non-HR (non-CO) pathways in older animals. Our data support defects in both DSB formation and in repair pathway choice.

We reasoned that if DSB efficiency did not decrease with age and DSB levels remained high, then to explain the observed decrease in RAD-51 foci, increasing numbers of chromosomes would be expected undergo non-HR repair. In fact, since most nuclei in day 4 adults have fewer than 10 RAD-51 foci (Figure 2B), this would mean that 10-20 DSBs would need to be repaired by alternative pathways. Not only would fewer bivalents be observed, but many larger fusions (and possibly fragments as well) would be seen at diakinesis. This is not what we observed. It was not until the cessation of reproductive life, that more than one fusion chromosome was observed (Figure 1A). Instead, > 90% of nuclei appear to repair DSBs as intact, bivalent-like or univalent-like chromosomes throughout the reproductive span. Thus, we infer that substantially fewer DSBs are made as the animal ages. In support of this conclusion, when we trapped RAD-51 protein on the ssDNA filaments with the *rad-54* mutation, RAD-51 levels were significantly reduced. Additionally, the preponderance of bivalent-like chromosomes in the older animals suggests that most chromosomes received a crossover despite low DSB levels, consistent with our previous data on CO distribution in aging animals (Lim et al., 2008). This data suggests that crossover homeostasis mechanisms (Martini et al., 2006) remain proficient in older animals. The reduction in DSBs that we observe either could be a result of an abrogation of SPO-11 activity or could reflect impairments in crossover feedback control mechanisms. The ATM-1 and ATR/ATL-1 kinases are activated by DSBs and downstream ssDNA intermediates and function in a regulatory feedback loop to retain DSB competency until a CO competent intermediate is achieved on all chromosomes (Stamper et al., 2013; Lukaszewicz et al., 2018; Li and Yanowitz, 2019). One possibility is that these signaling pathways could be impaired in aging animals to prevent the maintenance of DSB competency.

The increase in fusion chromosomes in aged diakinesis nuclei supports the notion that breaks are being diverted into non-HR repair pathways (Figure 1A). The impairment in recruitment of both RPA-1 and RAD-51 after IR and microirradiation also supports the notion that very early events in DSB repair are affected by age. In particular, these studies point to resection as the likely step in repair that is age-sensitive, as processing of the DSB ends regulates both the competition between repair pathways and the ability to recruit RPA-1 (Marini et al., 2019). Chromosome fusions result from repair through NHEJ, MMEJ, or SSA. On day 1 of adulthood, HIM-5 and CEP-1/p53 proteins redundantly inhibit NHEJ during meiosis (Meneely et al., 2012). Although the CO defects of *him-5* worsen with maternal age (Meneely et al., 2012), chromosome fusions were not observed, suggesting that the CEP-1 dependent block to NHEJ is still intact in older animals. The proteins that prevent repair via MMEJ and SSA are currently unknown, so we can only postulate that one or both of these pathways may be accessed to induce the fusions see in aged nuclei.

HR-mediated repair is also severely compromised in aged germ cell nuclei. Using both γ-irradiation and microirradiation, we see significant deficits in the recruitment, accumulation, and turnover of both RPA-1 and RAD-51 at DSB sites (Figures 3–5). One caveat to these studies is that we do not know whether old and young worms’ chromosomes are equally responsive to irradiation. However, since the nuclear envelope integrity deteriorates (Haithcock et al., 2005; D’Angelo et al., 2009) and chromatin decompacts in older animals (reviewed in Purohit and Chaturvedi, 2017; Sławińska and Krupa, 2021), we would anticipate that DNA damage would increase rather than decrease in older nuclei (Hernández et al., 2015). Further studies on the accessibility of chromatin to DNA damage and developing assay to directly monitor breaks in this system are an avenue of future studies. This impairment is seen in nuclei from the mitotic tip through late pachytene suggesting it reflects an overall loss of germ line capabilities with age. While this study has been focused on meiotic DNA repair, we note that the effects observed in the mitotic tip of the worm germ line may elucidate how aging impacts repair in replicative cells, including but not limited to the mammalian male germ cells which are maintained by continuous mitotic divisions. Although the general trends between IR and microirradiation are remarkably similar, there are notable differences particularly in regard to RPA-1 accumulation and turnover (Figures 3, 5A). This suggests that the source of DNA damage can dramatically change the persistence of repair intermediates and possibly the outcomes of repair. Microirradiation results in more complex form of DNA damage (clustered DSBs) that are more challenging to repair than dispersed DSBs, like the ones formed by γ-irradiation (Nickoloff et al., 2020). If repair processes are more challenging in older worms, microirradiation may result in more severe effects on recruitment of repair proteins to DSBs, compared to γ-irradiation. Further studies will be required to understand the molecular differences between these repair pathways and how they deteriorate with maternal age.

In the oldest oocytes (>day 6), we observed a novel appearance of some bivalents where the distance between univalents was increased. This separation of the bivalent might occur if, like in mammals and flies, cohesion between exchange homologs is lost with maternal age (Hodges et al., 2005; Subramanian and Bickel, 2008). Additional studies will be required to determine whether cohesin proteins expression or turnover is indeed impacted by maternal age in *C. elegans* and whether there is a functional consequence for the proper segregation of these bivalents.

## Data Availability Statement

The raw data supporting the conclusions of this article will be made available by the authors, without undue reservation.

## Author Contributions

MR performed the studies in Figures 1–3, Supplementary Figures 1, 3, 4 with some assistance by JY. RB performed the studies in Figures 4, 5, and Supplementary Figure 2. All authors contributed to design and interpretation of experiments and the writing and editing of the manuscript.

## Conflict of Interest

The authors declare that the research was conducted in the absence of any commercial or financial relationships that could be construed as a potential conflict of interest.

## Publisher’s Note

All claims expressed in this article are solely those of the authors and do not necessarily represent those of their affiliated organizations, or those of the publisher, the editors and the reviewers. Any product that may be evaluated in this article, or claim that may be made by its manufacturer, is not guaranteed or endorsed by the publisher.

## Supplementary Material

Supplementary Figure 1 RAD-51 staining in dissected germ lines from *spo-11(ok79)* mutants at 1 and 4 days post-L4. DNA is visualized with DAPI (green); RAD-51 with anti-RAD-51 antibodies (Magenta). Shown are maximum projection images through half of the germ line. Almost no RAD-51 foci were observed in germ lines from day 1 adults; approximately half of day 4 adult germ line pachytene nuclei had a single RAD-51 focus.

Supplementary Figure 2 GFP::RPA-1 levels are not diminished in aging *C. elegans* germline nuclei. **(A)** Representative images of pachytene nuclei from each age of worms examined expressing the endogenously- tagged GFP::RPA-1 fusion protein. Scale bars are 3 μm. **(B)** Western blot analysis of RPA-1 protein in 1 day-old and 4 day-old adults. OLLAS::RPA-1 fusion proteins were detected with anti-OLLAS antibodies. Tubulin serves as loading control. **(C–E)** Average intensity of GFP::RPA-1 in meiotic nuclei, corrected for cytoplasmic background. Each point indicates a single nucleus, error bars indicate the Standard Error of the Mean. **(C)** Pre-meiotic phase nuclei. **(D)** Transition zone (polarized chromatin, leptotene/zygotene phases) nuclei. **(E)** Mid- and late-pachytene phase nuclei. Statistical significance was determined using the Mann-Whitney *U*-test and *p*-values are indicated as follows: ns ≥ 0.1234, 0.0332–0.1233 = ^∗^0.0021–0.0331 = ^∗∗^0.0002–0.0020 = ^∗∗∗^ ≤ 0.0001 = ^****^. Nuclei *n*-values as indicated for each condition.

Supplementary Figure 3 Percentage of middle pachytene (MP) nuclei containing the indicated number (key at far right) of GFP::RPA-1 foci (left) or RAD-51 foci (right) in *spo-11* at timepoints post-10Gy of γ-irradiation. Numbers on top of bars indicate the total number of nuclei analyzed per age. A total of 10 worms per age/timepoint were analyzed. Statistical significance was determined using Chi-square test. ns *p* > 0.1, ^∗^*p* < 0.05, ^****^*p* < 0.0001.

Supplementary Figure 4 Quantification of DAPI-bodies in *cku-70* and *polq-1* diakinesis nuclei from the indicated ages. Data analyzed in Figure 3 for N2 worms of the same ages was added to allow the comparison with the mutants. Numbers on top of bars indicate the total number of nuclei analyzed per age.
