# Supplementary figures and images for: Aging Negatively Impacts DNA Repair and Bivalent Formation in the C. elegans Germ Line
Source: Front Cell Dev Biol. 2021 Aug 4;9:695333. doi: 10.3389/fcell.2021.695333 (PMC8371636; doi:10.3389/fcell.2021.695333)

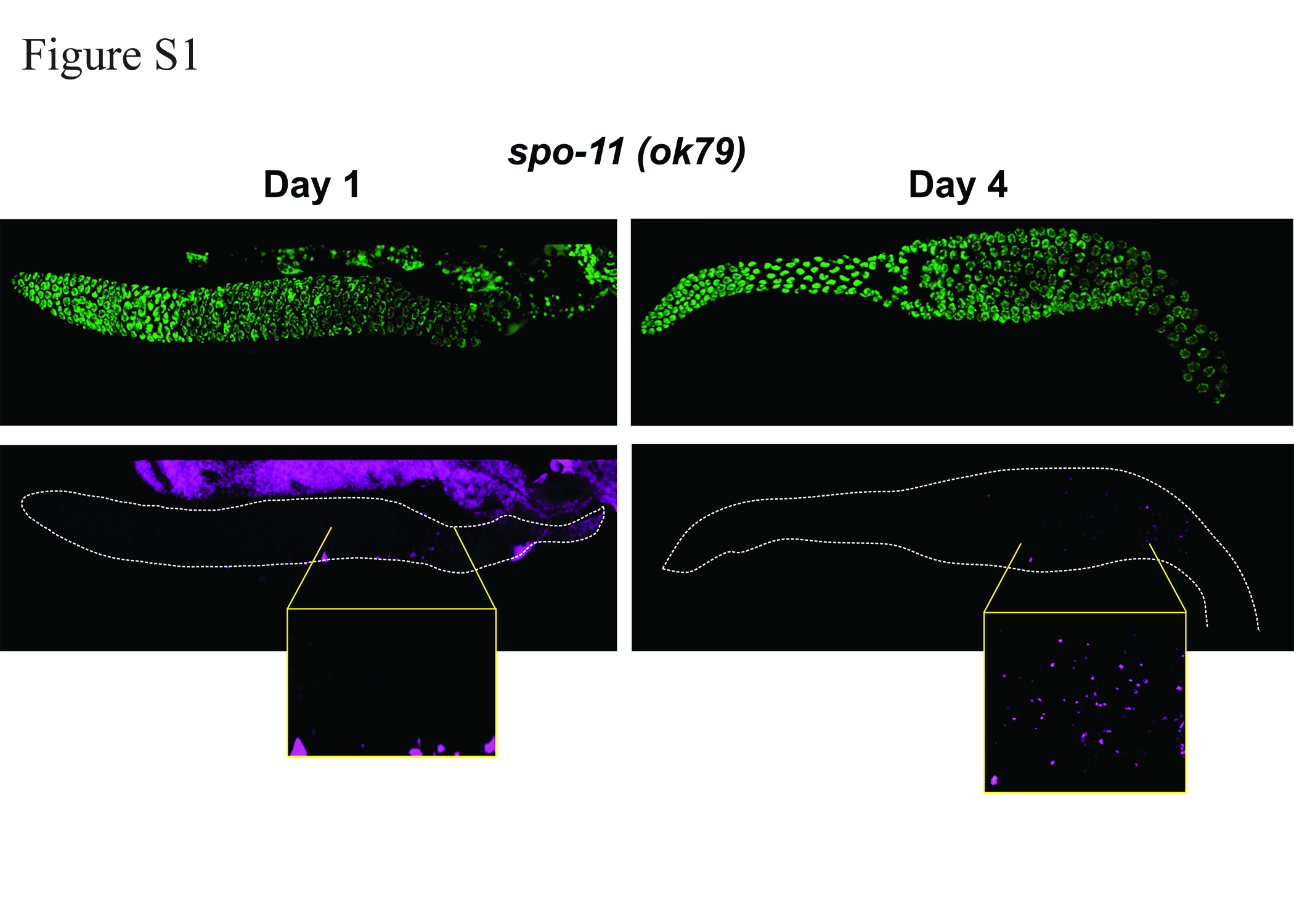

Supplement: Supplementary Figure 1 — RAD-51 staining in dissected germ lines from spo-11(ok79) mutants at 1 and 4 days post-L4. DNA is visualized with DAPI (green); RAD-51 with anti-RAD-51 antibodies (Magenta). Shown are maximum projection images through half of the germ line. Almost no RAD-51 foci were observed in germ lines from day 1 adults; approximately half of day 4 adult germ line pachytene nuclei had a single RAD-51 focus. [file Image_1.TIF]

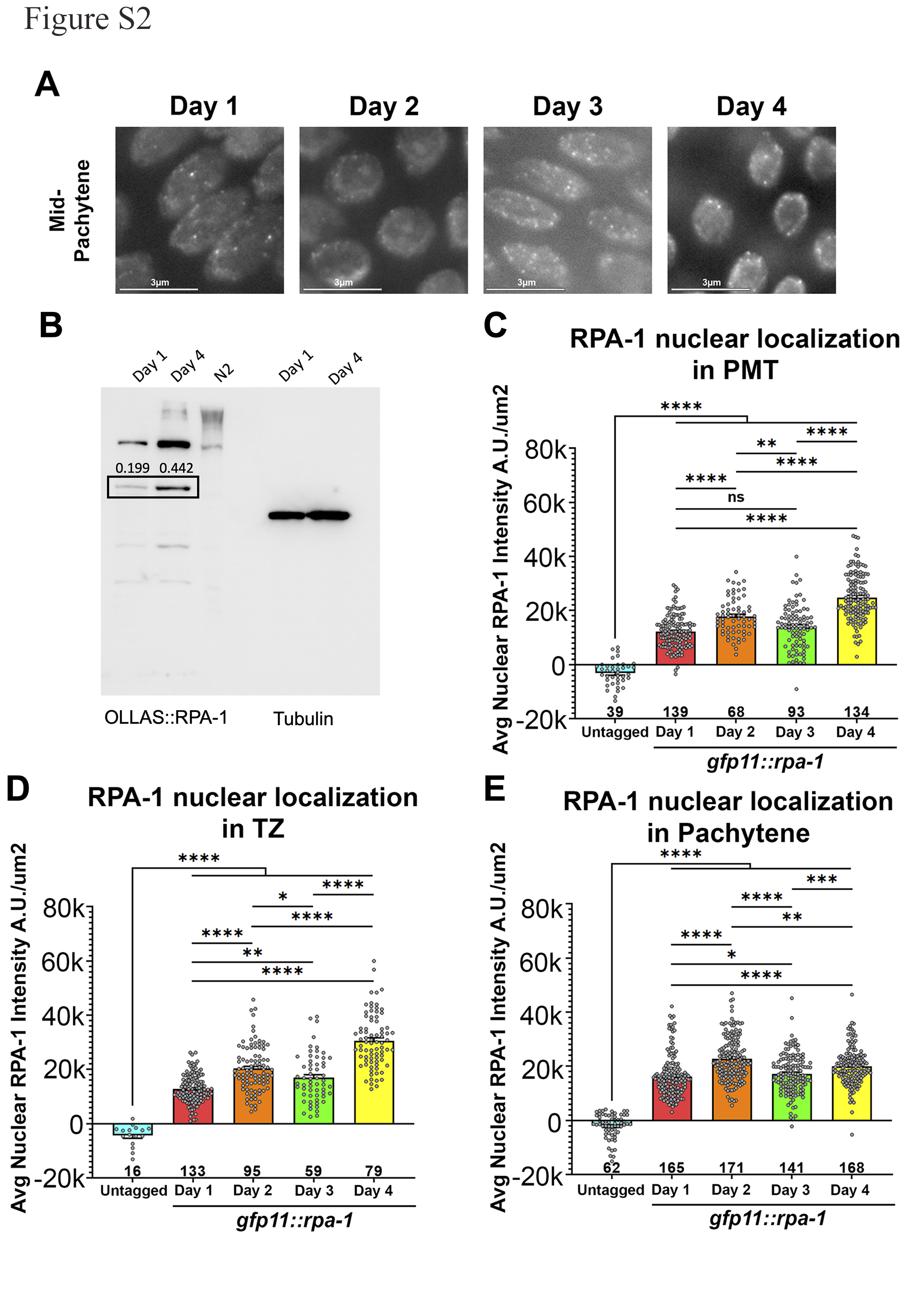

Supplement: Supplementary Figure 2 — GFP::RPA-1 levels are not diminished in aging C. elegans germline nuclei. (A) Representative images of pachytene nuclei from each age of worms examined expressing the endogenously- tagged GFP::RPA-1 fusion protein. Scale bars are 3 μm. (B) Western blot analysis of RPA-1 protein in 1 day-old and 4 day-old adults. OLLAS::RPA-1 fusion proteins were detected with anti-OLLAS antibodies. Tubulin serves as loading control. (C–E) Average intensity of GFP::RPA-1 in meiotic nuclei, corrected for cytoplasmic background. Each point indicates a single nucleus, error bars indicate the Standard Error of the Mean. (C) Pre-meiotic phase nuclei. (D) Transition zone (polarized chromatin, leptotene/zygotene phases) nuclei. (E) Mid- and late-pachytene phase nuclei. Statistical significance was determined using the Mann-Whitney U-test and p-values are indicated as follows: ns ≥ 0.1234, 0.0332–0.1233 = ∗0.0021–0.0331 = ∗∗0.0002–0.0020 = ∗∗∗ ≤ 0.0001 = ****. Nuclei n-values as indicated for each condition. [file Image_2.TIF]

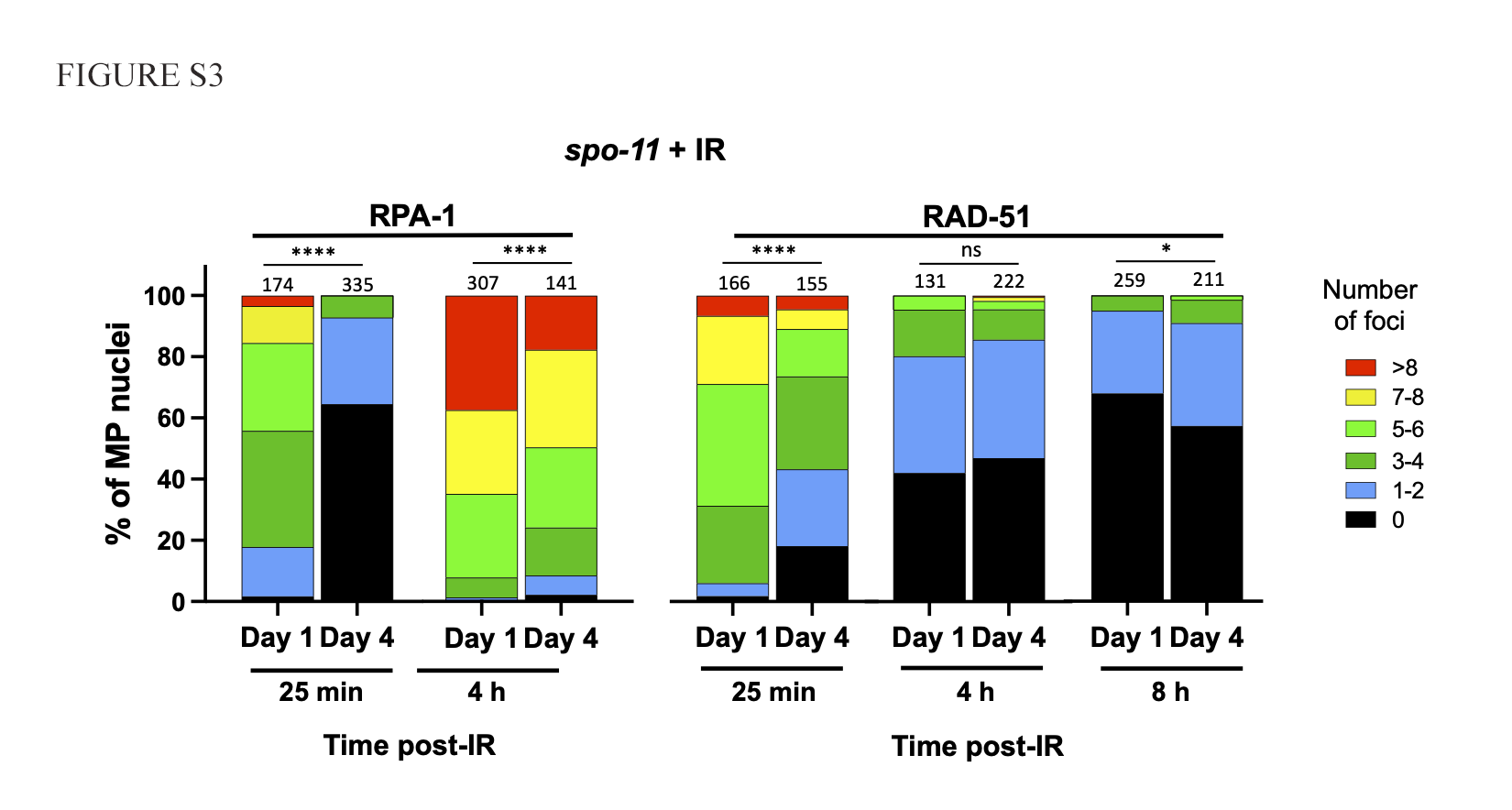

Supplement: Supplementary Figure 3 — Percentage of middle pachytene (MP) nuclei containing the indicated number (key at far right) of GFP::RPA-1 foci (left) or RAD-51 foci (right) in spo-11 at timepoints post-10Gy of γ-irradiation. Numbers on top of bars indicate the total number of nuclei analyzed per age. A total of 10 worms per age/timepoint were analyzed. Statistical significance was determined using Chi-square test. ns p > 0.1, ∗p < 0.05, ****p < 0.0001. [file Image_3.TIFF]

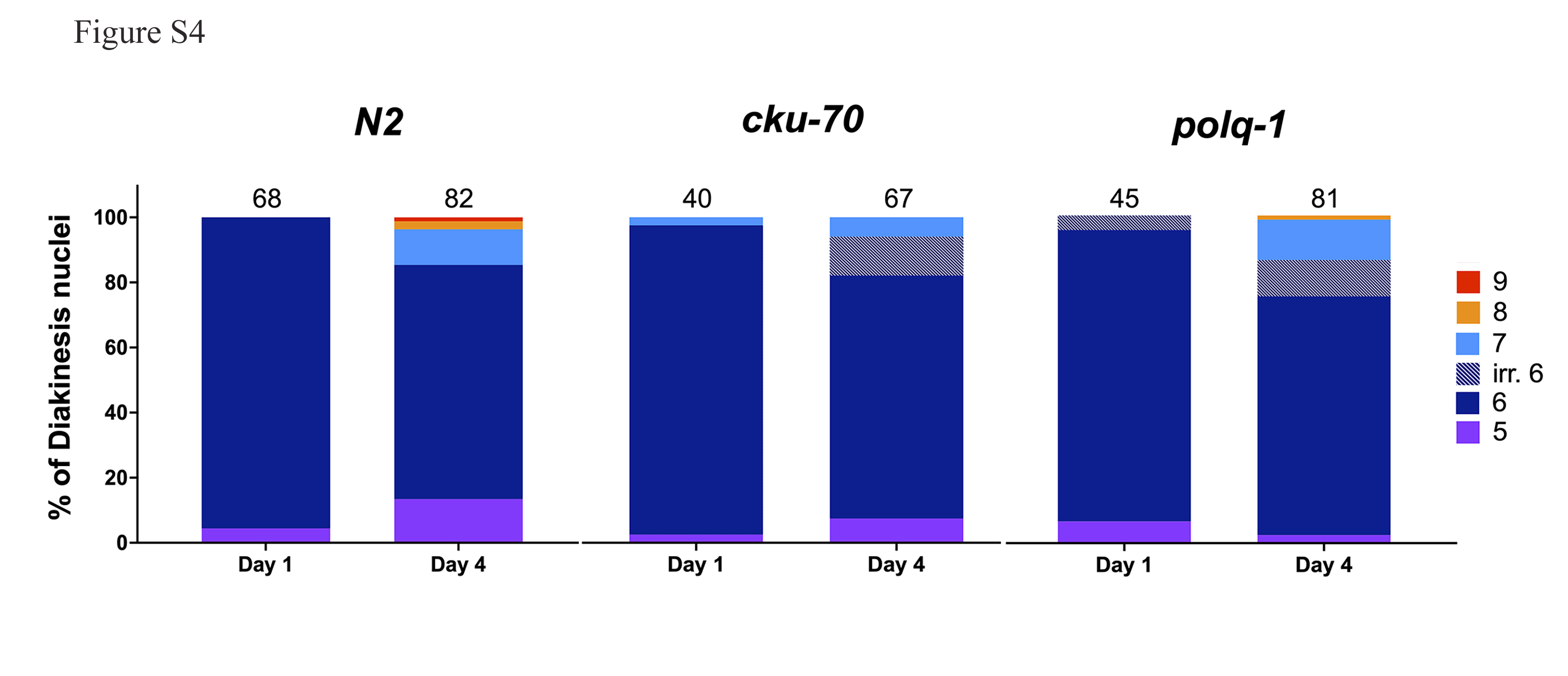

Supplement: Supplementary Figure 4 — Quantification of DAPI-bodies in cku-70 and polq-1 diakinesis nuclei from the indicated ages. Data analyzed in Figure 3 for N2 worms of the same ages was added to allow the comparison with the mutants. Numbers on top of bars indicate the total number of nuclei analyzed per age. [file Image_4.TIF]
